# A review of the current state of the computer-aided diagnosis (CAD) systems for breast cancer diagnosis

**DOI:** 10.1515/biol-2022-0517

**Published:** 2022-12-09

**Authors:** Zicheng Guo, Jiping Xie, Yi Wan, Min Zhang, Liang Qiao, Jiaxuan Yu, Sijing Chen, Bingxin Li, Yongqiang Yao

**Affiliations:** Department of Breast and Thyroid Surgery, Affiliated Zhongshan Hospital of Dalian University, No. 6, Jiefang Road, Dalian City, 116001, China

**Keywords:** breast cancer, mammography, computer-aided diagnosis, ultrasound

## Abstract

Breast cancer is one of the most common cancers affecting females worldwide. Early detection and diagnosis of breast cancer may aid in timely treatment, reducing the mortality rate to a great extent. To diagnose breast cancer, computer-aided diagnosis (CAD) systems employ a variety of imaging modalities such as mammography, computerized tomography, magnetic resonance imaging, ultrasound, and histological imaging. CAD and breast-imaging specialists are in high demand for early detection and diagnosis. This system has the potential to enhance the partiality of traditional histopathological image analysis. This review aims to highlight the recent advancements and the current state of CAD systems for breast cancer detection using different modalities.

## 1 Introduction

Breast cancer is becoming the most frequent cancer among women, and if it is detected during the first stage, it has a good chance of being cured. Thus, early recognition of breast cancer is crucial for its effective treatment [1]. The ducts that carry milk to the nipple or in the glands that produce breast milk is the region where breast cancer usually starts [2]. From the American Cancer Society’s forecasts for 2019, there will be about 268,600 new cases of invasive breast cancer in women in the United States, about 62,930 new noninvasive cases, and about 41,760 deaths from breast cancer [2]. It is becoming the most essential health distress among women worldwide, which accounts for about 22–27% of different types of cancer [3]. However, early detection, screening, and intervention aid in minimizing the mortality rate and detection even before symptoms [4]. Artificial intelligence (AI) has no boundaries in the present day and is changing and saving lives. AI serves the purpose of developing and facilitating the provider and patient interactions. AI employs the simulation of human intelligence, notably computer systems, and comprises the ability to study and resolve problems [5,6]. In health care, technology and AI are enhancing their applications in decision support, image analysis, and patient triage. The concept of computer-aided diagnosis (CAD) was first described by Winsberg in 1967. When making a diagnosis, CAD uses pattern recognition software that distinguishes unfamiliar forms in the image for the physician to consider [7]. Different imaging modalities such as mammography (MM), ultrasonography (USG), computerized tomography (CT), magnetic resonance imaging (MRI), and biopsy are employed by CAD systems for breast cancer diagnosis. CAD also enhances the interpretation competence and analytical routine of radiologists by saving reading time and preserving the steadiness of the lesion recognition. In breast imaging, machine learning has been applied using CAD [8]. It serves as a substantiate or “second pair of eyes,” thereby substituting the second reading or observation by another radiologist. CAD also helps in interpreting and processing correct medical images, thereby highlighting conspicuous parts [8,9]. CAD is mostly intended to reduce human error from observations and false reports when reading images. It is well established that computers play an essential role in assisting radiologists in acquiring data management and reporting medical images for MRI, CT, and USG [10]. The current level of performance for CAD systems is encouraging, but not sufficient to make CAD systems standalone detection and diagnosis clinical systems [2]. Since 2007, research articles from the Multiple Breast Cancer Surveillance Consortium have revealed that CAD reduces performance by increasing recalls and decreasing the detection of invasive cancer while increasing the detection of ductal carcinoma *in situ* [8]. Various CAD systems that aid in the early detection of breast cancer have three stages. These include the detection of tumors, segmentation, and classification based on the shape of the tumor and subtypes using deep learning models. The initial stage of detection is based on the region of interest (ROI), which identifies the mammographic picture of a tumor using a faster convolutional neural network detector [11]. The algorithms of CAD systems mostly rely on mammograms, and to overcome the dependence on the operator and to enhance the precision of diagnosis rate, there are established breast cancer recognition and grouping images available [12]. Despite the wide and varied developments that CAD has attained since the beginning of the computer era, certain challenges are still faced by CAD systems [13]. Numerous algorithmic limitations, input data assembly, preprocessing, processing, and system assessments are challenges in CAD procedures. Algorithms are normally designed to choose a single likely diagnosis, thus providing suboptimal reports for patients with multiple concurrent disorders [14]. The effective design implementation and analysis of electronic health records are the main requirements of CAD systems [13]. The main stages of the CAD system are presented in Table 1.

**Table 1:** Main stages of CAD system

Stages	Function
Image preprocessing stage	To remove noise and defect caused in image acquisition procedure, image resizing, and enhance the image intensity
Segmentation	*Discontinuity-based segmentation:* Partitions an image based on an abrupt change in intensity
	*Similarity-based segmentation*: Partitions an image according to pre-determined similarity criteria. The similarity-based method is categorized into region-based, thresholding-based, and clustering-based methods
Feature extraction	Different features are extracted according to the characteristics of lesions from the image
	These features are used to distinguish benign or malignant lesions. The feature set is usually very large and the selection of the most effective features is very critical for the next step
1 Classification	According to the selected features, the suspicious areas are classified to benign or malignant based on different classification
2 Result	Diagnosis result
3 Performance evaluation	This step evaluates the performance of CAD system

## 2 Facilitators and barriers to CAD’s use

The frequency of breast cancer detection has gradually improved because of the use of CAD facilitators compared with double reading which saves time and expediency in breast cancer imaging [15]. However, the barriers to CAD provide less promising sensitivity, thereby increasing the rate of recall, higher outlay, and undefined effects on patient results [16]. Therefore, there is a need for meaningful research on the implementation and barriers to the use of CAD using clinical data analysis [15]. While considering the barriers against facilitators, the cost-effectiveness of the extensive use of CAD in MM must be properly evaluated before investing in it [16]. A high number of incorrect positive marks is one of the main drawbacks of CAD. The false-positive marks confused the radiologists and potentially lengthened the reading time. As with any technology, CAD implementation is costly and may not always be cost-effective [15]. There was a significant elongation in the reading time with CAD use [17]. Regarding CAD usefulness, explanation time increased marginally in another study but was not significantly developed using an interactive CAD system [18,19].

## 3 Mammographic reading

The Image Checker system was the first Food and Drug Administration (FDA)-approved CAD MM in 1998 for commercial use, and later, various systems were approved, including CAD systems for breast MRI, colon cancer, and lung cancer for analyzing MM [20]. Recently, CAD systems have been used for diagnostic assistance to improve physician’s medical decision-making [20]. MM is a low-dose X-ray that allows radiologists to observe any obvious changes in the breast tissue and is a devoted imaging modality for breast screening [12]. It is presently the most successful tool to detect breast cancer at an early stage when treatment is most likely to be successful. In mammographic inspection, non-cancerous lesions can be misunderstood because of human error or type-1 error, which may be overlooked with false negatives [12]. In dense breasts, there is a higher chance of under-rate during MM, in which the likelihood of cancer is 4–6 times higher than that in non-dense breasts [21]. MM has a sensitivity of 76.5% and a specificity of 87.1%, which makes it a good modality for decreasing breast cancer-related deaths in women younger than 40 years [22]. While screening MM, which is good quality for detecting actual cases, has some drawbacks with a greater chance of type-I error and false positives, which may cause some unnecessary biopsies due to low specificity [23]. Estimations of the risk of breast cancer linked with mammographic density may be distorted as density impacts the detection of cancer [21]. Risk may be undervalued if it is based exclusively on cancers found at screening, as cancers disguised by dense tissue will be omitted [21]. To further explore suspicious areas, a diagnostic mammogram is usually suggested if there is an abnormality in screening breast tissues. The process of screening mammograms is monotonous work that is very tiring and causes asthenopia because a radiologist must analyze more than 400–500 cases per day. In fact, a radiologist may detect 4–5 cancer cases out of every 400–500 cases and there is a high chance of human error in noticing images [24]. A test with low specificity in screening MM may yield a high number of false positives [23]. The mammograms were read in sets of approximately 120, with equal numbers of randomly well-organized case patients and control subjects, by the same observer, who was oblivious of the case or control position or of the classifications finished by the radiologists [21]. In a mammogram of a woman with invasive ductal carcinoma, seven of the nine readers correctly localized cancer, but rated their finding substantially more suspicious in the session with interactive CAD enabled, one reader only located cancer correctly in the session where CAD was enabled, and one reader assigned a slightly lower rating to cancer in the session with CAD [18]. To identify and label suspicious regions, hybrid methods are also based on independent component analysis (ICA), fuzzy classifiers, etc. [24]. Differential analysis was performed to evaluate the mammographic images of the present patient to discover any doubtful masses that may have altered its morphology [2]. Recent advances in AI technology have facilitated the growth of CAD systems with experimentally proven records, assisting radiologists in addressing the task of interpreting mammographic images [25]. Before a tumor becomes palpable and invasive, MM can detect it and it has become the most commonly used screening modality [4]. CAD and its success in conventional X-ray MM have inspired research on automated diagnostic techniques in breast MRI for further investigative and screening events [1]. MM has proven favorable correctness, with a single radiologist interpreting 25–30% of visible cancers [26]. Radiologists usually evaluate the marks after assembling their individual explanations and relate them to extend the final valuation of the image [8]. Factors such as partial pattern marking and physical boundaries such as asthenopia and stress usually hamper the human interpretation of mammograms with higher chances of human error. Moreover, low-quality images and noise with reduced visibility can obstruct radiologists and CAD interpretations [27]. Radiologists explained that features from mammograms and sonograms were used as contributions for linear discriminant analysis and artificial neural network (ANN) models to differentiate benign from malignant lesions [23]. Digital mammograms are scanned by CAD, and distrustful areas of breast cancer masses and microcalcifications are marked [8]. Rao et al. reported that private offices (81%) used more CAD compared to hospitals (70%) for breast cancer screening [28]. There was a slight increase in MM screening size by 2% from 2004 to 2008; however, there was an increase in the utilization of CAD system screening by approximately 90%. The IARC reported that there is a 23% decrease in breast cancer death rates using mammographic screening programs after estimating data from 40 collective reports [29,30].

## 4 Ultrasound

**Figure 1:**
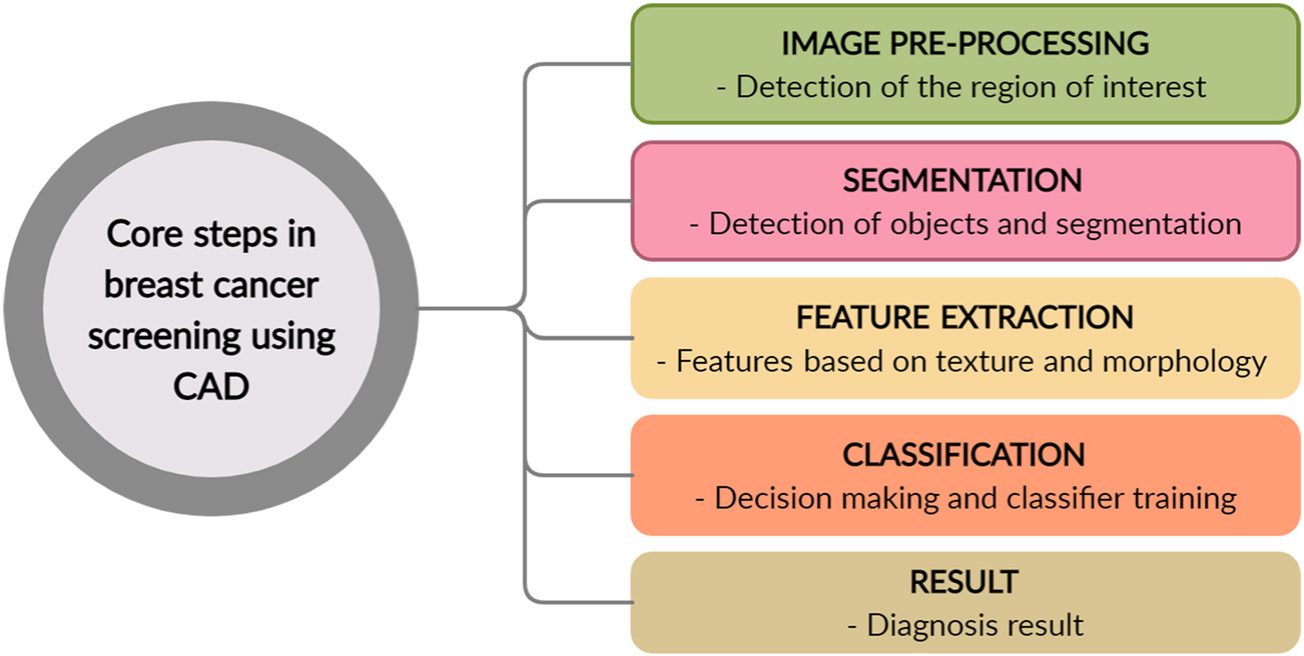
Core steps employed in breast cancer screening with the help of CAD systems.

Ultrasound is a valuable modality for assessing breast issues and monitoring findings in physical examinations and MM [12]. Ultrasound detects and differentiates benign tumors from malignant irregular borders with great accuracy and decreases the number of needless biopsies [31,32]. USG is also preferred among lactating and pregnant women for breast cancer screening. Although MRI is more sensitive than ultrasound, ultrasound has emerged as an important device for assisting mammograms owing to its superior options [12]. Ultrasound is dependent on the operator and its interpretation requires advanced knowledge from the user or radiologist [12]. In dense breasts, ultrasound has better sensitivity for detecting aggressive cancer [33]. In comparison with nonmalignant tumors, CAD systems and software normally showed higher efficiency in detecting lesions [34]. Numerous CAD schemes have been proposed for characterizing malignant and benign breast lesions on ultrasound scans. Hadjiiski et al. considered a CAD system with a combination of high-frequency sound waves and low-dose X-ray MM and assessed approximately 100 patients with lesions using this system [35]. The encouraging outcome of CAD results for MM has encouraged various radiologists and medical professionals to explore the potential application of CAD for understanding breast cancer-related images [35]. Fleury et al. studied to analyze the applicability of the strain elastography system for breast masses classified based on US diagnosis and counted based on the measure projected by imaging [36]. The US elastography results prior to biopsy as deduced by radiologists using a CAD system for a strain found that the intra-class association was 0.67 coefficient between different observers without CAD and 0.81 with a CAD system for strain elastography. The CAD system for strain elastography has certain prospects for enhancing its diagnostic performance for breast cancer inspection by ultrasound connected with elastography [36]. CAD reduces the number of extra biopsies on the breast; these systems help achieve biopsies of suspicious breast lesions seen on a mammogram with and without the aid of a computer-aided diagnostic system for strain elastography. Ultrasound elastography images before biopsy were interpreted by three radiologists, and the parameters evaluated by each radiologist were sensitivity, specificity, and diagnostic accuracy [36]. Ultrasound with elastography allows the assessment of stiffness of the ROI and assumes that malignant lesions are harder [36]. The block diagram of the CAD system involved in breast cancer diagnosis using USG is presented in Figure 1.

## 5 MRI

MRI illustrates the size of lobular breast cancer tumors more precisely than mammograms or ultrasound scans [37]. For nearly 30 years, the use of MRI has been engaged in the identification of lesions for breast cancer and its diagnostics [38]. The elucidation procedure of MRI seems to be laborious and necessitates employing highly experienced radiologists to identify benign and malignant lesions [39]. Breast cancer can also be detected in stage I in women who have undergone extraordinary menace in tracking the disease [37]. MRI for use in medical investigations has delivered an enormous on-ward jump in the field of diagnosis, particularly with exposure avoidance to potentially hazardous ionizing radiation [37]. The CAD system handles huge images that present an angioma with a multicolor picture that can relate to the kinetic features of a breast lesion. Contrasting and diagnostic lesion specificity can also be improved by using CAD. One of the drawbacks of MRI is that the evaluation of breast cancer requires a significant amount of time for image processing and interpretation. In addition, inter- and intra-observer variations are supplementary drawbacks of breast MRI. A large number of CAD systems are being established for varying breast-imaging modalities [40]. CAD for MRI is used to distinguish as (1) noninvasive and invasive breast lesions, (2) invasive cancers without lymph node (LN) metastasis, and (3) invasive breast cancers with LN metastasis. A few clinical reports have demonstrated the use of CAD for MRI as a method of tumor staging. The test for the significance of the size differences among the imaging methods was performed using the Kruskal–Wallis test. The observed *p*-value of 0.165 indicated no significant difference in size among the six imaging techniques. Its use of MRI is prevalent among clinical practitioners because of its availability and cost-efficiency. Nottingham and Aberdeen pioneered the production of clinical imaging MRI in 1980. The technique is currently a powerful and widely available clinical tool [40,41]. Since its first clinical use in the 1980s, MRI has become a progressively valuable musculoskeletal diagnostic tool, aiding in staging and monitoring treatment, in addition to its current diverse applications and future potential [42]. It provides a dynamic noninvasive evaluation of organs, cartilage, and muscles, in different planes (two or three-dimensional) [43]. MRI is highly valuable in the identification and observation of multiple musculoskeletal and neuromuscular conditions in the complete age spectrum owing to the lack of ionizing radiation in MRI [43].

With decreasing costs and better availability, its use is becoming ever more worldwide throughout clinical practice, and MR spectroscopy is also considered with an overview of key metabolites and how they have to be interpreted [40]. The perception of MRI as an important tool with promising potential can be achieved through a clinician’s understanding of the principles of the tool. The amplification of MRI systems, associated with higher field strengths and new sequences, has great diagnostic and treatment planning potential in musculoskeletal conditions [43,44]. Considering the principles of imaging modality and its numerous applications, it can be used to appreciate the benefits and limitations of its use, further updating clinical decision-making [40].

MRI can be performed to assess breast health as follows: (1) screening for breast cancer in people with a higher risk of developing the disease, (2) identification of tumors and metastasis in patients with breast cancer, (3) examination of tumor reappearance following surgical or chemotherapeutic treatment, and (4) screening for ruptured implants.

## 6 Biopsy

Traditional methods, such as tissue biopsy, are not efficient enough for cancer detection because of their inability to capture the whole genomic topography of tumors [45]. A tissue biopsy can provide a definitive diagnosis after analysis by a pathologist. Biopsies were classified based on the size of the needles used to collect tissue samples [37]. Breast cancer diagnostic tools are dependent on radiological and clinical evaluations supported by histopathological observations.

Radiomics works by extracting quantitatively distinct characteristics of cancer from radiological data, whereas liquid biopsy extracts the complete biology of malignancy from a blood sample [46]. Various components of tumor cells released into the blood circulation can be analyzed by liquid biopsy sampling, some of which include circulating tumor cells (CTCs), circulating tumor DNA (ctDNA), cell-free RNA, tumor-educated platelets, and exosomes. These components can be used for different purposes [45]. Lesions with more than 2% possibility of malignancy are recommended for biopsy to reduce false-negative diagnostic results [47]. Therefore, only 15–30% of patients undergoing biopsy are diagnosed with malignancy [47]. The novel technology is expected to make diagnostic and prognostic identifications of breast cancer with a potential reduction in the need for complex invasive biopsies, with a personalized approach [46]. Liquid biopsy sampling can be used to analyze different components of tumor cells in the blood circulation, including ctDNA, CTCs, cell-free RNA, and exosome tumor-educated platelets [45]. Computational diagnosis has escalated the diagnostic procedure, allowing extensive screening [46]. Nuclei segmentation is one of the major challenges in automatically analyzing cytological images using computer-aided methods. Kowal et al. performed a study to test and compare four different clustering algorithms in fast nuclei segmentation [48]. They found that CAD detection performance was independent of tumor histopathology and cancer size and that the CAD system accurately marked a vast majority of breast cancers proven using biopsy sampling [35,49]. Many studies have proven CAD as a feasible method for the classification of benign and malignant breast lesions using contrast-enhanced MRI [35]. Nattkemper et al. developed a classifier to differentiate malignant and benign breast lesions using MRI data from 74 cases (25 benign and 49 malignant) [50]. In a medical decision support system for breast cancer diagnosis, the cases were classified as either benign or malignant wherein the segmented nuclei and 42 morphological, topological, and texture features were extracted and used in a classification procedure with three different classifiers [48]. They utilized dynamic contrast-enhanced MRI data to extract contour and wash-out attributes that were resolved by radiologists. They obtained an Az of 0.88 using a support vector machine (SVM) or SVM classifier [35]. Increasing applications of MRI and USG are being evaluated. With the introduction of novel techniques, the application of liquid biopsy has been enhanced, allowing the improvement of numerous aspects of breast cancer control with early diagnosis and screening, prediction of prognosis, early detection of relapse, serial sampling and efficient longitudinal monitoring of disease progression, and response to treatment [45]. Most clinical applications are currently assigned to the detection of breast cancer and CAD characterization is expected to be a crucial component of next-generation CAD systems [35].

## 7 CT scan

CT scans were used to examine the spread of breast cancer to other organs of the body. However, this scanning technique is ineffective in the early stages of breast cancer. In advanced stages of breast cancer, CT scans are used to verify the cancer response to screening [37]. A CT scan works by engaging numerous narrow beams of X-rays over a specific body site that provides a multi-dimensional view of the patient’s body. The 3D structural volume of the breast was determined using the obtained images. A CT scan uses X-rays to obtain comprehensive divisional images of the body. CT scanners take many pictures, unlike normal X-rays, which take one or two pictures and these pictures are integrated using a computer to visualize a segment of the body part being studied. CT scans can visualize tumors that cannot be located using conventional MM. However, a CT scan is not usually used to check breasts but rather used to check whether tumor has spread or not. This test is commonly used to visualize the chest and abdominal regions to check for the metastasis of breast cancer, such as the liver or lungs. In a positron emission tomography (PET) scan, partially radioactive sugar [18-fludeoxyglucose (FDG)] is administered into the bloodstream, which then accumulates in cancer cells. PET scans are used in combination with CT scans through a special machine that can perform both simultaneously, allowing the doctor to compare the regions of maximum radioactivity on the PET scan with a more comprehensive picture on the CT scan. Contrast-enhanced computed tomography functionalities include (1) determination of the advancement of the breast cancer stage to select a suitable treatment for breast conservation (BCT); (2) determination of the degree of resection after neoadjuvant chemotherapy, which is challenging to diagnose using other methods; (3) diagnosis of metastasis to the axillary lymph node; the specificity and sensitivity are 70–89 and 79–90%, respectively; and (4) identification of occult breast cancer, axillary metastasis, and reappearance after BCT. For massive breast cancer, CT is recommended to screen for metastasis to the chest wall. This assists in determining whether cancer can be removed by mastectomy [37]. Speed, comfort, static artifacts, effortless standardization, and vast applicability are important advantages of CT scans. A dynamic contrast-enhanced CT scan is effectively used to detect the intraductal expansion of breast carcinoma in breast cancer. It is also useful in the pre-operative examination of disease progression before breast-conserving surgery.

## 8 Generalized pseudo-Zernike moment (GPZM) diagnosis of breast cancer

Singh and Urooj used the characteristics of GPZM (Generalized pseudo-Zernike moments) and PZM (pseudo-Zernike moments) as an efficient texture descriptors for utilizing the suspicious portions in a mammogram [51]. The adaptive differential evolution wavelet neural network is an improved classifier with improved accuracy in CAD system classification. This system’s competence was evaluated in various mammograms and tested on the MIAS database, which observed a precise result of 0.89 having 0.935 in the area under the curve (95% CI = upper limit of 0.82 and lower limit of 0.98). This technique was further examined for in-plane rotation, which was found to be extremely rotary-motion invariant. Moreover, this classifier was inspected and compared to other popular techniques based on the receiver operating characteristic (ROC) analysis using the DDSM database as a reference. The proposed classifier has an improved area under the curve (AUC) of 0.93 with a high precision rate [51].

## 9 Combination of modalities

Different multimodal breast cancer imaging techniques, such as FDG-PET, MRI, mammography, CT, and USG, are used for the pre-operative examination of breast cancer staging. Aristokli et al. evaluated each distinct modality in a study and MRI was reported with an overall specificity and sensitivity of 74.2 and 94.6%, respectively, while the specificity and sensitivity of MM were 85.5 and 54.5% [52]. The specificity and sensitivity of ultrasound were 76.8 and 67.2%. In combination with the results of the different techniques, the resulted specificity was 63.3% and sensitivity was 97.7%. Sensitivity was found to increase further with the combination of MM + MRI, USG + MRI, or MRI + MM + USG. Diagnostic ability increases with the combination of the techniques as compared to diagnosis using the modalities alone. In the presence of contraindications to MRI, CE-MM could be a good alternative in dense breast tissue because of its high sensitivity based on breast cancer types [52]. The combination of ANNs and fuzzy logic in the form of a fuzzy adaptive learning control network–adaptive resonance theory (FALCON-AART) complementary learning fuzzy neural network (CLFNN) was a major improvement in CAD systems. The possibility of achieving an accuracy of 90% was attributed to the FALCON-AART-based CAD design. Therefore, CAD is a valuable tool for diagnosing breast cancer. The use of thermography in breast cancer centers can be globally promoted by the high performance and cost-effectiveness of accurate CAD designs combined with new infrared systems [53]. Wavelet-based contourlet transform (WBCT) is an improved feature-extraction technique used to extract the features of the ROI, with better accuracy than conventional approaches. An approach for hybrid feature selection was proposed to reduce the dimensions of the features. This approach combines SVM and a genetic algorithm (GA), aiming to select the best combination of tumor indicators and maximize the discriminative potential [54]. The greatest improvement in CAD systems was achieved with a combination of fuzzy logic and ANNs in the form of a FALCON-AART CLFNN [53]. The lower cost and high performance of new infrared systems combined with accurate CAD designs can promote the use of thermography in many breast cancer centers worldwide [53]. The results indicated that the proposed CAD system (WBCT + GA–SVM-mutual information + kernel SVM) was superior to other standard techniques with a classification accuracy of 97.5% for normal–abnormal and 96% for benign–malignant. This approach exhibits minimal computational requirements compared to other techniques [54]. The experimental conclusions on both acquired primary datasets showed that all classifier-level and deep learning-based feature-level learning using privileged information (PI) acquired additional PI modality algorithms. These can enhance the performance of single-modal-imaging-based CAD for breast cancer by repositioning PI [37,51].

## 10 Discussion

Owing to commercially available FDA-approved schemes, the main clinical usage of CAD is for screen-film MM [35]. A summary of work done on CAD is presented in Table 2. For revealing and analysis of breast tumors as a “second opinion” appraisal supplementing the radiologist’s report, CAD systems can be used [44]. Based on DCE-MRI built on a post hoc approach, which was trained using weakly explained data, a new method was proposed for breast cancer screening [1]. The advantages and disadvantages of both approaches, when applied to breast screening from DCE-MRI, were targeted to launch and found out that depending on experiments on a breast DCE-MRI dataset that contains scans of 117 patients, the results inferred that the post hoc method was more precise for diagnosing the whole volume per patient [1]. A new generation of CAD and diagnosis systems is being developed due to new and advanced studies, also, leveraging AI-driven tools to competently read breast tomosynthesis imaging as well as digital mammograms. The use of AI in computational radiology demands transparency and difficult testing [11]. It is a challenge for the medical imaging specialists to design robust and reliable CAD systems for these NME lesions; the diagnosis of mass augmentation lesions is straightforward and employs typical characteristic parameters such as speculation (morphology), rim enhancement (texture), and washout kinetics [44]. However, the diagnosis of foci and non-mass-like enhancing lesions poses a challenge to both clinical reading and CAD systems [44]. Seeing the single modalities, the highest sensitivity was observed for MRI and the lowest sensitivity for MM regardless of breast type, density, and history. It was observed that the sensitivity increased from the combination of US + MRI or MM + MRI or MRI + MM + US [52]. The superiority and accuracy of conventional CAD systems have been improved by the development of AI and AI-based algorithms. Conventional CAD systems are based on handcrafted features; as a subfield of AI, deep learning is based on representational learning [59]. This review provides an overview of CAD for the diagnosis and detection of breast cancer. Facilitators and barriers to CAD were also presented. Different stages of CAD are also presented in the present study such as the GPZM which acts as a well-organized texture descriptor of doubtful parts in a mammogram. Different CAD modalities and their specificity when using a single or a combination of modalities are also presented, which will provide good insight to clinicians while handling patient’s breast mass samples. This review provides information on various CAD systems that also serve as a basis for comparison between the most recent techniques.

**Table 2:** A summary of work done on CAD diagnosis

Article	Study objective	Results	Study conclusion	Contributor
Review article	(i) Adoption and implementation of CAD during breast cancer screening	(i) There is a trade off between the facilitators and barriers for CAD use	(i) The cost-effectiveness of CAD has not been well established for breast cancer screening in various populations	Masud et al., 2019 [15]
	(ii) to describe barriers and facilitators for CAD use	(ii) Facilitators for CAD use improved breast cancer detection rates, increased profitability of breast imaging, and time saved by replacing double reading	(ii) Research is needed on how to best facilitate CAD in radiology practices in order to optimize patient outcomes, and the views of radiologists	
Original research	(i) To evaluate the diagnostic performance of the CAD system in full-field digital MM for detecting breast cancer when used by dedicated breast radiologist (BR) and radiology resident (RR)	(i) Sensitivity improved with CAD use in the BR and RR groups (from 81.10 to 84.29% for BR and 75.38 to 77.95% for RR)	(i) CAD was helpful for dedicated BRs to improve their diagnostic performance and for RRs to improve the sensitivity in a screening setting	Jung et al., 2014 [19]
	(ii) To investigate the benefit of CAD application		(ii) CAD could be essential for radiologists by decreasing reading time without decreasing diagnostic performance	
Original research	(i) To evaluate a commercial tomosynthesis cCAD system in an independent, multicenter dataset	(i) Use of the CAD system showed per-lesion sensitivity of 89% (99 of 111; 95% confidence interval	(i) A digital breast tomosynthesis CAD system can allow detection of a large percentage of breast cancers manifesting as masses and microcalcification clusters, with an acceptable false-positive rate	Meyer-Base et al. 2021 [44]
		(ii) 62 of 72 lesions detected were masses	(ii) Further studies with larger datasets acquired with equipment from multi-parametric imaging and breast cancer radiomics	
		(iii) Overall, 37 of 39 microcalcification clusters (95% sensitivity, 95% confidence interval: 81%, 99%) and 79 of 89 masses (89% sensitivity, 95% confidence interval: 80%, 94%) were detected with the CAD system		
Original research	(i) To evaluate the value of the CAD program applied to diagnostic breast ultrasonography (US) based on operator experience	(i) Out of 100 breast masses, 41 (41%) were malignant and 59 (59%) were benign	(i) CAD is a useful additional diagnostic tool for breast US in all radiologists, with benefits differing depending on the radiologist’s level of experience	Park et al., 2019 [55]
		(ii) compared with the experienced radiologists, the less experienced radiologists had significantly improved negative predictive value (86.7–94.7% vs 53.3–76.2%, respectively)	(ii) CAD improved the inter-observer agreement and showed acceptable agreement in the characterization of breast masses	
		(iii) experienced radiologists had significantly improved specificity (52.5 and 54.2% vs 66.1 and 66.1%) and positive predictive value (55.6 and 58.5% vs 64.9 and 64.9%, respectively) with CAD assistance (all *P* < 0.05)		
Original research	To develop a breast CADx methodology that addresses the efficiency of pre-trained convolutional neural networks (CNNs) and using preexisting handcrafted CADx features	(i) From ROC analysis, the fusion-based method demonstrates, imaging modalities with statistical significant improvements	(i) A novel breast CADx methodology that can be used to more effectively characterize breast lesions in comparison to existing methods	Antropova et al., 2017 [56]
		(ii) AUC compared to previous breast cancer CADx methods in the task showed distinguishing result between malignant and benign lesions		
Original research	To analyze the cost-effectiveness of adding computer-aided detection (CAD) to a screening MM program	(i) Cost-effectiveness was expressed as the marginal cost per year of life saved (MCYLS)	(i) The cost-effectiveness of CAD is dependent on the magnitude of the increase in cancer detection rates with CAD	Lindfors et al., 2006 [57]
		(ii) CAD to a mammographic screening program resulted in a MCYLS of $19,058 and yields a linear increase in MCYLS	(ii) It is also affected by the stage distribution of cancers diagnosed with CAD	
			(iii) MCYLS is greater for CAD added to screening versus screening MM alone but is within the accepted cost-effective range	
Original research	(i) To investigate the efficacy of CAD for MRI in tumor extent, lymph node status, and multifocality breast cancers	(i) MRI with CAD had the highest area under the receiver operating characteristic curve (AUC = 0.888)	(i) CAD for breast MRI can be a feasible method of evaluating tumor extent and multifocality in invasive breast cancer patients	Song et al., 2015 [58]
	(ii) To compare CAD detection for MRI with other breast-imaging modalities			

## 11 Conclusions

The performance of breast cancer detection depends on (1) the performance of the CAD system (2) the population under application, and (3) the radiologists using the system. The use of CAD is beneficial for inexperienced radiologists for the detection of breast carcinomas present as microcalcifications. In the modern era, advancements in AI necessitate the knowledge of using technologies such as CAD, in the clinical setting, their impact on clinical practitioners, and their potentially changing roles. Further studies are needed to better understand CAD systems and discover better applications in healthcare settings. Evaluation of the cost of the CAD system used for breast carcinoma screening needs to be implemented to simplify the applications of CAD.

## References

[1] Maicas G, Bradley AP, Nascimento JC, Reid I, Carneiro G. Pre and post-hoc diagnosis and interpretation of malignancy from breast DCE-MRI. Med Image Anal. 2019;58:101562. 10.1016/j.media.2019.101562. [PMID: 31561184]. 31561184

[2] Ramadan SZ. Methods used in computer-aided diagnosis for breast cancer detection using mammograms: a review. J Healthc Eng. 2020;2020:9162464. 10.1155/2020/9162464. [PMID: 32300474]. PMC7091549 32300474

[3] Ferlay J, Soerjomataram I, Dikshit R, Eser S, Mathers C, Rebelo M, et al. Cancer incidence and mortality worldwide: sources, methods and major patterns in GLOBOCAN 2012. Int J Cancer. 2015;136(5):E359–86. 10.1002/ijc.29210. [PMID: 25220842]. 25220842

[4] Bahl M. Management of high-risk breast lesions. RadiolClin North Am. 2021;59(1):29–40. 10.1016/j.rcl.2020.08.005. [PMID: 33222998]. 33222998

[5] Dreyer KJ, Geis JR. When machines think: radiology’s next frontier. Radiology. 2017;285(3):713–8. 10.1148/radiol.2017171183. [PMID: 29155639]. 29155639

[6] Tang A, Tam R, Cadrin-Chênevert A, Guest W, Chong J, Barfett J, et al. Canadian association of radiologists white paper on artificial intelligence in radiology. Can Assoc Radiol J. 2018;69(2):120–35. 10.1016/j.carj.2018.02.002. [PMID: 29655580]. 29655580

[7] Fernandes F, Barbalho I, Barros D, Valentim R, Teixeira C, Henriques J, et al. Biomedical signals and machine learning in amyotrophic lateral sclerosis: a systematic review. Biomed Eng Online. 2021;20(1):61. 10.1186/s12938-021-00896-2. [PMID: 34130692]. PMC8207575 34130692

[8] Keen JD, Keen JM, Keen JE. Utilization of computer-aided detection for digital screening mammography in the united states, 2008 to 2016. J Am Coll Radiol. 2018;15(1 Pt A):44–8. 10.1016/j.jacr.2017.08.033. [PMID: 28993109]. 28993109

[9] Paquerault S, Hardy PT, Wersto N, Chen J, Smith RC. Investigation of optimal use of computer-aided detection systems: the role of the “machine” in decision making process. Acad Radiol. 2010;17(9):1112–21. 10.1016/j.acra.2010.04.010. [PMID: 20605489]. 20605489

[10] Castellino RA. Computer aided detection (CAD): an overview. Cancer Imaging. 2005;5(1):17–9. 10.1102/1470-7330.2005.0018. [PMID: 16154813]. PMC1665219 16154813

[11] Tran WT, Sadeghi-Naini A, Lu FI, Gandhi S, Meti N, Brackstone M, et al. Computational radiology in breast cancer screening and diagnosis using artificial intelligence. Can Assoc Radiol J. 2021;72(1):98–108. 10.1177/0846537120949974. [PMID: 32865001]. 32865001

[12] Jalalian A, Mashohor S, Mahmud R, Karasfi B, Saripan MIB, Ramli ARB. Foundation and methodologies in computer-aided diagnosis systems for breast cancer detection. EXCLI J. 2017;16:113–37. 10.17179/excli2016-701. [PMID: 28435432]. PMC5379115 28435432

[13] Tate JE, Burton AH, Boschi-Pinto C, Parashar UD. World Health Organization–Coordinated Global Rotavirus Surveillance Network. Global, regional, and national estimates of rotavirus mortality in children <5 years of age, 2000–2013. Clin Infect Dis. 2016;62:S96–S105. 10.1093/cid/civ1013. [PMID: 27059362]. PMC11979873 27059362

[14] Wadhwa RR, Park DY, Natowicz MR. The accuracy of computer-based diagnostic tools for the identification of concurrent genetic disorders. Am J Med Genet A. 2018;176(12):2704–9. 10.1002/ajmg.a.40651. [PMID: 30475443]. 30475443

[15] Masud R, Al-Rei M, Lokker C. Computer-aided detection for breast cancer screening in clinical settings: scoping review. JMIR Med Inform. 2019;7(3):e12660. 10.2196/12660, Erratum in: JMIR Med Inform. 2019; 7(3):e15799. [PMID: 31322128]. PMC6670274 31322128

[16] Guerriero C, Gillan MG, Cairns J, Wallis MG, Gilbert FJ. Is computer aided detection (CAD) cost effective in screening mammography? A model based on the CADET II study. BMC Health Serv Res. 2011;11:11. 10.1186/1472-6963-11-11. [PMID: 21241473]. PMC3032650 21241473

[17] Sohns C, Angic BC, Sossalla S, Konietschke F, Obenauer S. CAD in full-field digital mammography-influence of reader experience and application of CAD on interpretation of time. Clin Imaging. 2010;34(6):418–24. 10.1016/j.clinimag.2009.10.039. [PMID: 21092870]. 21092870

[18] Samulski M, Hupse R, Boetes C, Mus RD, den Heeten GJ, Karssemeijer N. Using computer-aided detection in mammography as a decision support. Eur Radiol. 2010;20(10):2323–30. 10.1007/s00330-010-1821-8. [PMID: 20532890]. PMC2940044 20532890

[19] Jung NY, Kang BJ, Kim HS, Cha ES, Lee JH, Park CS, et al. Who could benefit the most from using a computer-aided detection system in full-field digital mammography? World J SurgOncol. 2014;12:168. 10.1186/1477-7819-12-168. [PMID: 24885214]. PMC4046038 24885214

[20] Doi K. Computer-aided diagnosis in medical imaging: historical review, current status and future potential. Comput Med Imaging Graph. 2007;31(4–5):198–211. 10.1016/j.compmedimag.2007.02.002[PMID: 17349778] PMC1955762 17349778

[21] Boyd NF, Guo H, Martin LJ, Sun L, Stone J, Fishell E, et al. Mammographic density and the risk and detection of breast cancer. N Engl J Med. 2007;356(3):227–36. 10.1056/NEJMoa062790. [PMID: 17229950]. 17229950

[22] Nelson HD, Tyne K, Naik A, Bougatsos C, Chan B, Nygren P, et al. Screening for breast cancer: systematic evidence review update for the us preventive services task force. Rockville (MD): Agency for Healthcare Research and Quality (US); 2009. Report No.: 10-05142-EF-1. [PMID: 20722173]. 20722173

[23] Jesneck JL, Lo JY, Baker JA. Breast mass lesions: computer-aided diagnosis models with mammographic and sonographic descriptors. Radiology. 2007;244(2):390–8. 10.1148/radiol.2442060712. [PMID: 17562812]. 17562812

[24] Abu-Amara F, Abdel-Qader I. Hybrid mammogram classification using rough set and fuzzy classifier. Int J Biomed Imaging. 2009;2009:680508. 10.1155/2009/680508. [PMID: 19859576]. PMC2766069 19859576

[25] Tang X. The role of artificial intelligence in medical imaging research. BJR Open. 2019;2(1):20190031. 10.1259/bjro.20190031. [PMID: 33178962]. PMC7594889 33178962

[26] Noble M, Bruening W, Uhl S, Schoelles K. Computer-aided detection mammography for breast cancer screening: systematic review and meta-analysis. Arch Gynecol Obstet. 2009;279(6):881–90. 10.1007/s00404-008-0841-y. [PMID: 19023581]. 19023581

[27] Giger ML. Machine learning in medical imaging. J Am CollRadiol. 2018;15(3 Pt B):512–20. 10.1016/j.jacr.2017.12.028. [PMID: 29398494]. 29398494

[28] Rao VM, Levin DC, Parker L, Cavanaugh B, Frangos AJ, Sunshine JH. How widely is computer-aided detection used in screening and diagnostic mammography? J Am Coll Radiol. 2010;7(10):802–5. 10.1016/j.jacr.2010.05.019. [PMID: 20889111]. 20889111

[29] Lauby-Secretan B, Scoccianti C, Loomis D, Benbrahim-Tallaa L, Bouvard V, Bianchini F, et al. Breast-cancer screening--viewpoint of the IARC Working Group. N Engl J Med. 2015;372(24):2353–8. 10.1056/NEJMsr1504363. [PMID: 26039523]. 26039523

[30] Ragab DA, Sharkas M, Attallah O. Breast cancer diagnosis using an efficient cad system based on multiple classifiers. Diagnostics. 2019;9(4):165. 10.3390/diagnostics9040165. [PMID: 31717809]. PMC6963468 31717809

[31] Chen CM, Chou YH, Han KC, Hung GS, Tiu CM, Chiou HJ, et al. Breast lesions on sonograms: computer-aided diagnosis with nearly setting-independent features and artificial neural networks. Radiology. 2003;226(2):504–14. 10.1148/radiol.2262011843. [PMID: 12563146. 12563146

[32] Sahiner B, Chan HP, Roubidoux MA, Hadjiiski LM, Helvie MA, Paramagul C, et al. Malignant and benign breast masses on 3D US volumetric images: effect of computer-aided diagnosis on radiologist accuracy. Radiology. 2007;242(3):716–24. 10.1148/radiol.2423051464. [PMID: 17244717]. PMC2800986 17244717

[33] Costantini M, Belli P, Lombardi R, Franceschini G, Mulè A, Bonomo L. Characterization of solid breast masses: use of the sonographic breast imaging reporting and data system lexicon. J Ultrasound Med. 2006;25(5):649–59. 10.7863/jum.2006.25.5.649. [PMID: 16632790]. 16632790

[34] Chabi ML, Borget I, Ardiles R, Aboud G, Boussouar S, Vilar V, et al. Evaluation of the accuracy of a computer-aided diagnosis (CAD) system in breast ultrasound according to the radiologist’s experience. Acad Radiol. 2012;19(3):311–9. 10.1016/j.acra.2011.10.023. [PMID: 22310523]. 22310523

[35] Hadjiiski L, Sahiner B, Chan HP. Advances in computer-aided diagnosis for breast cancer. Curr Opin Obstet Gynecol. 2006;18(1):64–70. 10.1097/01.gco.0000192965.29449.da. [PMID: 16493263]. PMC2800983 16493263

[36] Fleury EFC, Gianini AC, Marcomini K, Oliveira V. The feasibility of classifying breast masses using a computer-assisted diagnosis (cad) system based on ultrasound elastography and BI-RADS Lexicon. Technol Cancer Res Treat. 2018;17:1533033818763461. 10.1177/1533033818763461. [PMID: 29551088]. PMC5882047 29551088

[37] Lakshmi IVJ, Padmavathamma M. Potential of cad using image mining techniques for breast cancer screening: a review. Int J Innov Eng and Tech. 2016;7(3):323–9.

[38] Heywang SH, Wolf A, Pruss E, Hilbertz T, Eiermann W, Permanetter W. MR imaging of the breast with Gd-DTPA: use and limitations. Radiology. 1989;171(1):95–103. 10.1148/radiology.171.1.2648479. [PMID: 2648479]. 2648479

[39] Meeuwis C, van de Ven SM, Stapper G, Fernandez Gallardo AM, van den Bosch MA, Mali WP, et al. Computer-aided detection (CAD) for breast MRI: evaluation of efficacy at 3.0 T. Eur Radiol. 2010;20(3):522–8. 10.1007/s00330-009-1573-5Epub 2009 Sep 2. [PMID: 19727750] PMC2822230 19727750

[40] Grover VP, Tognarelli JM, Crossey MM, Cox IJ, Taylor-Robinson SD, McPhail MJ. Magnetic resonance imaging: principles and techniques: lessons for clinicians. J Clin Exp Hepatol. 2015;5(3):246–55. 10.1016/j.jceh.2015.08.001. [PMID: 26628842]. PMC4632105 26628842

[41] Hawkes RC, Holland GN, Moore WS, Worthington BS. Nuclear magnetic resonance (NMR) tomography of the brain: a preliminary clinical assessment with demonstration of pathology. J Comput Assist Tomogr. 1980;4(5):577–86. 10.1097/00004728-198010000-00001. [PMID: 6967878]. 6967878

[42] Dean Deyle G. The role of MRI in musculoskeletal practice: a clinical perspective. J Man Manip Ther. 2011;19(3):152–61. 10.1179/2042618611Y.0000000009. [PMID: 22851878]. PMC3143009 22851878

[43] McMahon KL, Cowin G, Galloway G. Magnetic resonance imaging: the underlying principles. J Orthop Sports Phys Ther. 2011;41(11):806–19. 10.2519/jospt.2011.3576. [PMID: 21654095]. 21654095

[44] Meyer-Base A, Morra L, Tahmassebi A, Lobbes M, Meyer-Base U, Pinker K. AI-Enhanced diagnosis of challenging lesions in breast mri: a methodology and application primer. J Magn Reson Imaging. 2021;54(3):686–702. 10.1002/jmri.27332. [PMID: 32864782]. PMC8451829 32864782

[45] Alimirzaie S, Bagherzadeh M, Akbari MR. Liquid biopsy in breast cancer: A comprehensive review. Clin Genet. 2019;95(6):643–60. 10.1111/cge.13514. [PMID: 30671931]. 30671931

[46] Pesapane F, Suter MB, Rotili A, Penco S, Nigro O, Cremonesi M, et al. Will traditional biopsy be substituted by radiomics and liquid biopsy for breast cancer diagnosis and characterisation? Med Oncol. 2020;37(4):29. 10.1007/s12032-020-01353-1. [PMID: 32180032]. 32180032

[47] Sickles EA. Periodic mammographic follow-up of probably benign lesions: results in 3,184 consecutive cases. Radiology. 1991;179(2):463–8. 10.1148/radiology.179.2.2014293. [PMID: 2014293]. 2014293

[48] Kowal M, Filipczuk P, Obuchowicz A, Korbicz J, Monczak R. Computer-aided diagnosis of breast cancer based on fine needle biopsy microscopic images. Comput Biol Med. 2013;43(10):1563–72. 10.1016/j.compbiomed.2013.08.003. [PMID: 24034748]. 24034748

[49] Dromain C, Boyer B, Ferré R, Canale S, Delaloge S, Balleyguier C. Computed-aided diagnosis (CAD) in the detection of breast cancer. Eur J Radiol. 2013;82(3):417–23. 10.1016/j.ejrad.2012.03.005. [PMID: 22939365]. 22939365

[50] Nattkemper TW, Arnrich B, Lichte O, Timm W, Degenhard A, Pointon L, et al. UK MARIBS Breast Screening Study. Evaluation of radiological features for breast tumour classification in clinical screening with machine learning methods. Artif Intell Med. 2005;34(2):129–39. [PMID: 15894177]. 10.1016/j.artmed.2004.09.001 15894177

[51] Singh SP, Urooj S. An improved cad system for breast cancer diagnosis based on generalized pseudo-zernike moment and Ada-DEWNN classifier. J Med Syst. 2016;40(4):105. 10.1007/s10916-016-0454-0. [PMID: 26892455]. 26892455

[52] Aristokli N, Polycarpou I, Themistocleous SC, Sophocleous D, Mamais I. Comparison of the diagnostic performance of magnetic resonance imaging (MRI), ultrasound and mammography for detection of breast cancer based on tumor type, breast density and patient’s history: A review. Radiography. 2022;S1078-8174(22)00006-2. 10.1016/j.radi.2022.01.006. [PMID: 35148941]. 35148941

[53] Moghbel M, Mashohor S. A review of computer assisted detection/diagnosis (CAD) in breast thermography for breast cancer detection. Artif Intell Rev. 2013;39:305–13.

[54] Llobet R, Pollán M, Antón J, Miranda-García J, Casals M, Martínez I, et al. Semi-automated and fully automated mammographic density measurement and breast cancer risk prediction. Comput Methods Programs Biomed. 2014;116(2):105–15. 10.1016/j.cmpb.2014.01.021. [PMID: 24636804]. 24636804

[55] Park HJ, Kim SM, La Yun B, Jang M, Kim B, Jang JY, et al. A computer-aided diagnosis system using artificial intelligence for the diagnosis and characterization of breast masses on ultrasound: Added value for the inexperienced breast radiologist. Medicine. 2019;98(3):e14146. 10.1097/MD.0000000000014146. [PMID: 30653149]. PMC6370030 30653149

[56] Antropova N, Huynh BQ, Giger ML. A deep feature fusion methodology for breast cancer diagnosis demonstrated on three imaging modality datasets. Med Phys. 2017;44(10):5162–71. 10.1002/mp.12453. [PMID: 28681390]. PMC5646225 28681390

[57] Lindfors KK, McGahan MC, Rosenquist CJ, Hurlock GS. Computer-aided detection of breast cancer: a cost-effectiveness study. Radiology. 2006;239(3):710–7. 10.1148/radiol.2392050670. [PMID: 16569787]. 16569787

[58] Song SE, Seo BK, Cho KR, Woo OH, Son GS, Kim C, et al. Computer-aided detection (CAD) system for breast MRI in assessment of local tumor extent, nodal status, and multifocality of invasive breast cancers: preliminary study. Cancer Imaging. 2015;15(1):1. 10.1186/s40644-015-0036-2. [PMID: 25888983]. PMC4344797 25888983

[59] Lucija B, Tatjana M. Application of CAD in the diagnosis of breast cancer. Radioloskivjesnik. 2022;46:2–11. 10.55378/rv.46.1.1.

